# SUBTLEX-CH: Chinese Word and Character Frequencies Based on Film Subtitles

**DOI:** 10.1371/journal.pone.0010729

**Published:** 2010-06-02

**Authors:** Qing Cai, Marc Brysbaert

**Affiliations:** Department of Experimental Psychology, Ghent University, Ghent, Belgium; University of Barcelona, Spain

## Abstract

**Background:**

Word frequency is the most important variable in language research. However, despite the growing interest in the Chinese language, there are only a few sources of word frequency measures available to researchers, and the quality is less than what researchers in other languages are used to.

**Methodology:**

Following recent work by New, Brysbaert, and colleagues in English, French and Dutch, we assembled a database of word and character frequencies based on a corpus of film and television subtitles (46.8 million characters, 33.5 million words). In line with what has been found in the other languages, the new word and character frequencies explain significantly more of the variance in Chinese word naming and lexical decision performance than measures based on written texts.

**Conclusions:**

Our results confirm that word frequencies based on subtitles are a good estimate of daily language exposure and capture much of the variance in word processing efficiency. In addition, our database is the first to include information about the contextual diversity of the words and to provide good frequency estimates for multi-character words and the different syntactic roles in which the words are used. The word frequencies are freely available for research purposes.

## Introduction

Research on the Chinese language is becoming an important theme in psycholinguistics. Not only is Chinese one of the most widely spoken languages in the world, it also differs in interesting ways from the alphabetic writing systems used in the Western world. For example, the logographic writing system makes it impossible to compute the word's phonology on the basis of non-lexical letter to sound conversions [1]. Another characteristic of the Chinese writing system is that there are no spaces between the words. This is likely to have consequences for eye movement control in reading [2]. Finally, a Chinese character represents a syllable, which most of the time is a morpheme (i.e., the smallest meaningful element), and many Chinese words in fact are disyllabic compound words [3].

Research on the Chinese language requires reliable information about word characteristics, so that the stimulus materials can be manipulated and controlled properly. By far the most important word feature is word frequency. In this text, we first describe the frequency measures that are available for Chinese. Then, we describe the contribution a new frequency measure based on film subtitles is making in other languages and we present a similar database for Mandarin Chinese.

### Available sources of Chinese word frequencies

A first way to find information about Chinese word frequencies is to look them up in published frequency-based dictionaries. The source most frequently used thus far has been the *Dictionary of Modern Chinese Frequency* ≪
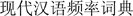
≫ (1986) [4], which is based on a corpus of 1.8 million characters (or 1.3 million words after segmentation) and provides frequency information for 31,159 words. Although this dictionary has been very useful, it is becoming increasingly outdated, as it is based on publications from the 1940s to the 1970s. A further limitation is the rather small size of the underlying corpus. Another dictionary that can be used is the *Frequency Dictionary of Modern Chinese words in common uses* ≪
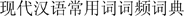
≫ (1990) [5]. This dictionary is based on a corpus of 25 millions characters, but unfortunately only provides information about the 10,000 most frequent words, making it less suited for low-frequency items. Most of other frequency-based dictionaries contain even less words. For instance, the recently published *A Frequency Dictionary of Mandarin Chinese: Core Vocabulary for Learners* [6] only contains information about the 5,000 most frequently used words.

A second source of word frequency information consists of frequency lists that have been compiled by linguists and official organizations ([7] for an earlier review). Most of these lists are not publicly available, but can be obtained from the researchers. In Table 1 we summarize the most interesting lists we have encountered in our search.

**Table 1:** Word frequency lists of Chinese.

The *Language Corpus System of Modern Chinese Study* (LCSMCS) word frequencies, based on a corpus of 20 million characters of which 2 million have been segmented into words and assigned their parts-of-speech (PoS) [8]; available at http://www.dwhyyjzx.com/cgi-bin/yuliao/, checked on September 24, 2009).
The *Center for Chinese Linguistics* (CCL) character frequency list, based on a corpus of Modern Chinese of 307 million characters, published by Peking University (for more information, see http://ccl.pku.edu.cn:8080/ccl_corpus/CCL_CC_Sta_Xiandai.pdf, checked on September 24, 2009).
The *Lancaster Corpus of Mandarin Chinese* (LCMC), based on a corpus of 73 million characters (50 million words; see http://www.lancs.ac.uk/fass/projects/corpus/LCMC/, checked on September 24, 2009). This is the corpus underlying *A frequency dictionary of mandarin Chinese: Core vocabulary for learners* [6].
The *Academia Sinica Balanced Corpus of Modern Chinese* based on 5 million characters and compiled by the Institute of Information Science and the CKIP group in Academia Sinica (http://www.sinica.edu.tw/SinicaCorpus/, checked on September 24, 2009).
*Draft for modern Chinese word set for common use* ≪ 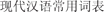 ≫ ( 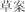 ) (2008) compiled by the *State Language Commission of China* [9]. This list contains 56,008 frequency-ranked words, the frequencies of which are based on a segmented part of 45 million characters from the *Chinese (General) Balanced Corpus*, a segmented corpus of 135 million characters based on *People's Daily 2001-2005*, and a modern Chinese literature corpus of 70 million characters constructed by Xiamen University. The word frequencies themselves, however, are not yet publicly available.
Word list *YW2001* (92,843 words) reported by Sun et al. [10] as part of the major outcome of the national corpus project by the *Chinese Information Processing Platform*, based on a corpus of ca. 800 million characters, but the list is not publicly accessible so far.

When reading Table 1, it is important to keep in mind that many corpora were meant to be representative for the language produced in Chinese speaking regions and not necessarily for the language daily heard and read by Chinese speaking people. In addition, some of these sources are copyright protected. One main problem with Chinese word frequencies is that Chinese words are not written separately, making the segmentation of the corpus into words labor-intensive if one wants to have information beyond single character frequencies (Chinese words can consist of one to four or even more characters). This situation is currently changing, due to the availability of automatic parsers and part-of-speech taggers, as we will see below.

All in all, despite the existence of several frequency lists in Chinese, there are only three sources that provide easy access for individual researchers and other people interested in the Chinese language. The first is *CCL* (http://ccl.pku.edu.cn:8080/ccl_corpus), which gives access to the unsegmented and untagged corpus and provides information about character frequencies but not word frequencies. The second is *LCSMCS* (http://www.dwhyyjzx.com/cgi-bin/yuliao/), which gives word frequencies based on the segmented part of the corpus (2 million words). Unfortunately, words have to be entered separately on the website. Part of the single-character word frequencies from LCSMCS are also available in the *Chinese Single-character Word Database* (CSWD; available at http://www.personal.psu.edu/pul8/psylin_norm/psychnorms.html). This database provides information about 2,390 single-character Chinese words including nouns, verbs, and adjectives [11]). Finally, there is the Lancaster Corpus of Mandarin Chinese (http://www.lancs.ac.uk/fass/projects/corpus/LCMC/) which provides frequency information for 5,000 words in *A frequency dictionary of mandarin Chinese: Core vocabulary for learners* [3] and for a larger set of 50,000 words upon request from the authors (also released by Richard Xiao on http://www.corpus4u.org).

### Subtitles as a valid source of word frequencies

Recent work by New, Brysbaert, and colleagues has indicated that film and television subtitles form a source of word frequencies that is more valid than the traditional books-based counts [12]–[14]. In particular, New et al. [14] showed that a corpus of several million words coming from thousands of popular films and television series can be obtained from websites specialized in providing subtitles for DVDs (in DVDs the subtitles form a separate channel that is superimposed on the film channel, so that subtitles can be made available in different languages). In addition, New et al. [14] showed that in French word frequencies based on film subtitles correlate more with word processing times (obtained from lexical decision tasks) than word frequencies based on books or internet pages.

Brysbaert and New [12] showed that the French findings are valid for English as well. First, they collected a corpus of 50 million words coming from nearly 9,000 different films and television sitcoms. Then they correlated the resulting word frequencies with the word naming times and the lexical decision times from the Elexicon project [15]. Brysbaert and New [12] found that subtitle frequencies not only explained more of the variance in naming times and lexical decision times than the other measures, but in addition they observed that a corpus of 16–30 million words was enough to have good frequency estimates. They also found that a measure based on contextual diversity (i.e., in how many films a word is used) was slightly better than the raw frequencies of occurrence, in line with a recommendation made by Adelman, Brown, & Quesada [16].

Keuleers et al. [13] reported essentially the same findings in Dutch. Their subtitle frequency measure, based on a corpus of 40 million words, explained nearly 10% more variance in lexical decision times (based on 14,000 monosyllabic and disyllabic words) than the existing golden standard, the Celex frequencies [17], [18].

Encouraged by the above findings, we decided to compile a word and character frequency list based on Chinese subtitles. A potential problem in this work is that, unlike in most writing systems, there are no spaces between the words in Chinese. Therefore, word segmentation (i.e. splitting the character sequence into words) is a critical step in collecting Chinese word frequencies. Fortunately, in the last decade automatic word segmentation programs have become available with a good output [for a review see 3]. These algorithms are trained on a tagged corpus (i.e., a corpus in which all the words have been identified and given their correct syntactic role) and are then applied to new materials [19]. Their performance is regularly compared in competitions such as the SIGHAN Bakeoff (www.sighan.org; SIGHAN: a Special Interest Group of the Association for Computational Linguistics). A program that consistently performed among the best is ICTCLAS (http://www.ictclas.org) [3]. It incorporates part-of-speech information (PoS, i.e. the syntactic roles of the words, such as noun, verb, adjective, etc.) and generates multiple hidden Markov models, from which the one with the highest probability is selected [19], [20]. This not only provides the correct segmentation for the vast majority of sentences, but also has the advantage that the most likely syntactic roles of the words are given, which makes it possible to additionally calculate PoS-dependent frequencies. The algorithm is expected to work well for film subtitles, because these subtitles are of a limited syntactic complexity (most of them are short, simple sentences) and because the program has the faculty to recognize out-of-vocabulary words such as foreign names, which often exist in subtitles but are rarely covered by regular vocabularies. The program was also used to parse the LCMC corpus.

We further calculated the frequency of occurrence of the characters (CHR), irrespective of whether they came from single-character words or from multi-character words. Character frequencies are interesting, because there is some evidence that characters in multi-character words contribute to the processing times of single-character words (see below) and because the word segmentation sometimes is ambiguous, with different readers making different interpretations, for instance in the context of compound words [21]–[23]. Something similar would exist in English, where some compounds are written as single words (flowerpot, football, honeymoon) and others not (flower seeds, foot locker, honey hive). If there were no spaces as external cues, it would be difficult to know how best to split these words.

Next to the word and character frequency measures (i.e. the number of times a word or a character occurs in the corpus), we also calculated the contextual diversity (CD) measure for the words and the characters. This is defined as the number of films in which the word or character appears. Extensive analyses by Adelman et al. [16] suggest that CD is a (slightly) more informative measure, a finding confirmed by Brysbaert and New [12] and Keuleers et al. [13]. We did not calculate the CD measure for the PoS dependent frequencies, as to our knowledge this information has not yet been needed.

All in all, five new frequency measures were calculated for Mandarin Chinese: Character frequency based on subtitles, character contextual diversity based on subtitles, word frequency based on subtitles, word contextual diversity based on subtitles, and word PoS-dependent word frequency based on subtitles. The three measures that go beyond the individual characters are particularly new and have been made possible due to the development of a reliable automatic PoS tagger.

To check the usefulness of the new frequency measures relative to the existing ones, we used third-party behavioral data to examine how well the different indices predicted word processing times. We also ran a new small-scale study, specifically aimed at testing the relative merits of text-based and subtitle-based frequencies for two-character words.

## Materials and Methods

### Corpus collection

Subtitle files are independent of the corresponding video files. They can either be extracted from existing DVDs or translated from the movie itself or subtitles available in other languages. The translation is usually done by highly proficient bilinguals (selected volunteers working as member of a ‘subtitle group’) and usually double-checked before they are published on the Internet. We got permission to download all the subtitle files from two of the biggest websites in China mainland providing subtitles in Simplified Chinese, by making use of GNU Wget (a Web crawler). We only retained the subtitle files in text-based SRT format and excluded all files in VobSub format, because the latter are image-based and require an additional optical character recognition (OCR) process to convert them into text (which certainly for Chinese characters is very error prone and needs to be proofread by humans).

To avoid the inclusion of double files (which could be the same file named differently or the same film translated by different people) and to identify files with technical errors (e.g., bad translations), all files were checked both automatically (for doubles) and manually by a Chinese native speaker. They were also properly coded, for instance to ensure that we knew which files belonged to the same film or television episode (as one film or episode may be divided into several subtitle files). This left us with 6,243 different contexts (7,148 files), about half of them coming from movies and half from television series. CD measures, namely the number of different contexts in which a word appeared, were calculated based on this.

For each subtitle file, the time zone information and other information not related to the film contents were removed (e.g., the name of the subtitle group, translator, proofreader, director, actors, etc.). The files were then segmented and PoS tagged with the ICTCLAS software (http://www.ictclas.org, Institute of Computing Technology, Chinese Lexical Analysis System [19]). We used the ICTCLAS version 2009Share via Java Native Interface (JNI). Regarding the PoS specifications, we used the Peiking University (PKU) PoS tagging set [24], [25] among the sets available in ICTCLAS. According to a previous study [26], this combination (PKU-ICTCLAS) has an excellent performance in word segmentation. The outcome of the analysis was a corpus of 33.5 million words (46.8 million characters).

### Calculation of the frequency measures

For each file the output of ICTCLAS provided us with lines of words (both single-character and multi-character words) and their part-of-speech (e.g. ‘
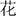
’ used as a verb [meaning spend/cost], a noun [meaning flower], or an adjective [meaning colorful]). We introduced some basic cleaning by removing non-Chinese characters included in low-frequency sequences, except for person names. For instance, the output ‘
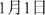
’ (meaning the 1st of January) was split and only ‘
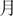
’ (month) and ‘
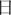
’ (date) retained.

As indicated above, five different frequency measures were calculated on the basis of the ICTCLAS output. These are made available in three easy-to-use files.

The first file (SUBTLEX-CH-CHR) includes the information about the characters. There were 5,936 different characters in the corpus. For each character we calculated the frequency based on total count (CHRCount) and based on CD (CHR-CD).

**Figure 1:**
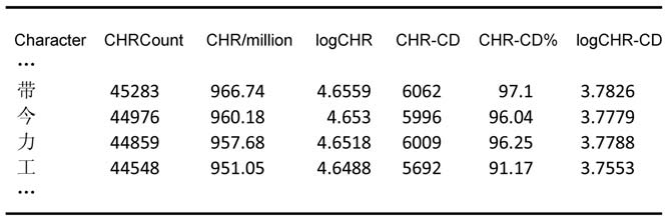
Lay-out of the SUBTLEX-CH-CHR file.

Figure 1 shows the lay-out of the information. The different columns are easy to interpret:

- **Character**: gives the character; ordered according to character frequency in Figure 1.
- **CHRCount**: shows the total number of times the character has been observed in the corpus (on a total of 46.8 million characters).
- **CHR/million**: provides the character frequency per million (with two digit precision not to lose information). This is the best value to report in manuscripts as it gives an idea of the character frequency independent of the corpus size.
- **logCHR**: is the log10 of CHRCount. Together with logCHR-CD, this is the best variable to match stimuli on if they have to be controlled for frequency.
- **CHR-CD**: is the number of films in which the character was observed (on a maximum of 6,243).
- **CHR-CD%**: is the percentage of films in which the character is observed. Because of the standardization, this measure is again independent of the corpus size.
- **logCHR-CD**: is the log10 of CHR-CD. Together with logCHR, this is the variable to match stimuli on if they have to be controlled for frequency.

**Figure 2:**
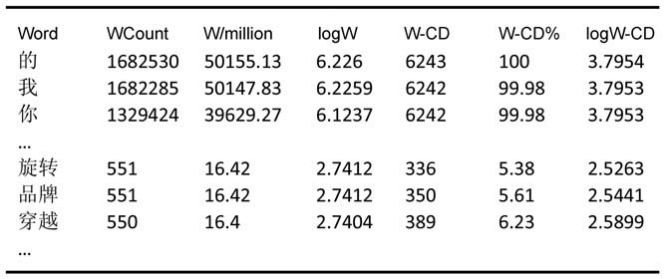
Lay-out of the SUBTLEX-CH-WF file.

The second file (SUBTLEX-CH-WF) contains the word form frequencies, both the numbers counted and the CDs. In total, our corpus included 99,121 different words. Figure 2 shows the lay-out of the information, which is analogous to that of the character frequencies (except for the fact that WCount is based on total of 33.5 million words).

**Figure 3:**
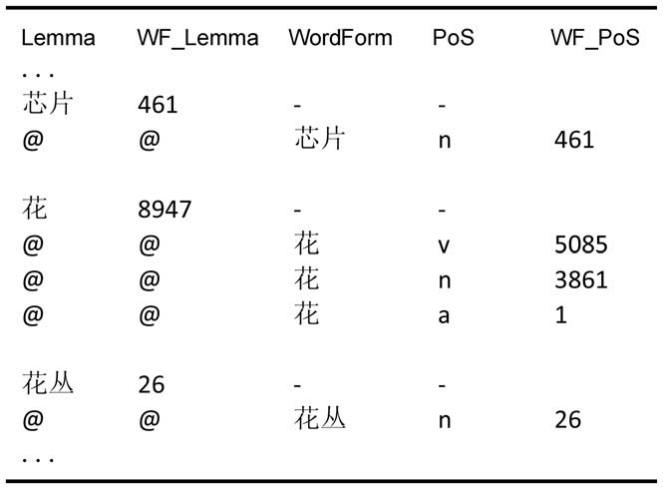
Lay-out of SUBTLEX-CH-WF_PoS file.

Finally, there is the SUBTLEX-CH-WF_PoS file, which contains information about the frequencies of the different syntactic roles words play. The layout of this file is kept similar to the frequency list of the British National Corpus (http://ucrel.lancs.ac.uk/bncfreq). Figure 3 gives an example of this file.

A line of the SUBTLEX-CH-WF_PoS file starting with a word signifies a lemma (e.g. the word‘
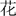
’ as a verb, as a noun, or as an adjective), and it is followed by the number of times it has been observed in the corpus (WF, with the same interpretation as WCount). Lines starting with ‘@→@’ signify all the possible PoS-roles associated with the lemma. The PoS label is given next. This is based on the PKU PoS system, and labels used in this system are listed in Table S1. Finally, the numbers of observations (WF_PoS) of the different syntactic roles are given, which sum up to the WF of the lemma.

### Validation of the new frequencies

The best way to validate word frequencies is to check how well they account for behavioral data. When we (M.B.) first calculated subtitle frequencies, we did not expect them to do particularly well, because criticisms can be raised against films as a representative source of language (they often depict American situations, are biased towards certain topics such as police investigations, do not include everything that is said, the language is not completely spontaneous, etc.). We just thought that they might tap into a language register (spoken television language) that was complementary to that of books. It was only when we saw how well these word frequencies were doing to predict word processing times for thousands of words [12], [13] that we started to appreciate their potential. Despite their shortcomings, subtitle frequencies are a very good indication of how long participants need to recognize words. They also better predict which words will be known to the participants and which not.

There are two reasons why the findings in English, French, and Dutch might not generalize to Chinese. First, the cultural differences between the setting of the film and the environment of the participants may be larger (i.e. a large proportion of the movies and TV shows popular in China are either American or European), making film subtitles less representative for daily life in China than in the Western world. Second, there is the issue of the segmentation. Although the outcome of the program looked good when it was checked by a native Chinese speaker, it is possible that some biases were still present.

We were able to get the data from two previously published studies (kindly provided to us by the authors). The first consisted of the naming latencies for 2,423 visually presented single-character words, collected by Liu [27], [ see also 11]. This database should give us good information about the usefulness of the frequency measures for single-character words, which have formed the stimulus materials for the majority of word recognition studies in Chinese so far.

To assess the validity of our frequencies, we compared them to 4 other measures. The first two were word frequency measures (i.e., only the frequencies of the characters used as separate words). They were LCSMCS and LCMC. The last two were character frequencies (i.e., the frequencies of the characters independent of whether they came from single-character words or from multi-character words). They came from LCSMCS (kindly sent to us by Ping Li) and CCL. To these measures we compared our own four measures: two word frequency indices (SUBTL_logW and SUBTL_logW-CD) and two character frequency indices (SUBTL_logCHR, and SUBTL_logCHR-CD).

A first thing we observed was that not all the characters used by Liu [27] had word frequencies. There were 37 missing observations (i.e. 1.5%) in our SUBLEX-CH-WF database, 68 missing observations (2.8%) in the LCSMCS word database, and 58 missing observations (2.4%) in the LCMC database). A scrutiny by native speakers suggested that some characters were indeed very rare as single-character words but were well-known constituents of two-character words (for example, 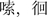
are regularly used as the second characters of words but rarely as words themselves in modern Chinese). In order not to favor one or the other database, we ran the correlation and regression analyses on the 2,289 words that were covered by all frequency measures. The frequencies were log10 transformed.

Table 2 shows the intercorrelations between the different measures. From this table, it can be seen that the correlations between our word frequencies and the LCSMSC word frequencies are .791 for SUBTL_logW and .786 for SUBTL_logW-CD. The correlations between our word frequencies and the LCMC word frequencies are .854 for SUBTL_logW and .840 for SUBTL_logW-CD. These are in line with the correlations observed between subtitles and written frequencies in the other languages tested (English, French and Dutch; unpublished data). The correlation table also shows the significant negative correlations between the naming RTs and each of the frequency measures, with higher correlations for the character frequencies than for the word frequencies.

**Table 2:** The intercorrelations between RT and eight available frequency measures (four word frequency measures and four character frequency measures) for 2289 single character words in a word naming task.

N = 2289		RT	Word frequency				Character frequency			
			SUBTL_logW	SUBTL_ logW-CD	LCSMCS_logW	LCMC_logW	SUBTL_logCHR	SUBTL_logCHR-CD	LCSMCS_logCHR	CCL_ logCHR
RT		1								
Word frequency	SUBTL_ logW	−.532*	1							
	SUBTL_ logW-CD	−.533*	.979*	1						
	LCSMCS_logW	−.479*	.791*	.786*	1					
	LCMC_ logW	−.548*	.854*	.840*	.877*	1				
Character frequency	SUBTL_ logCHR	−.566*	.825*	.819*	.666*	.770*	1			
	SUBTL_ logCHR-CD	−.571*	.780*	.806*	.617*	.721*	.970*	1		
	LCSMCS_logCHR	−.559*	.687*	.678*	.728*	.796*	.855*	.822*	1	
	CCL_ logCHR	−.547*	.670*	.657*	.660*	.778*	.866*	.831*	.962*	1

*p<0.01.

To further test the merits of the different frequency measures, we ran multiple regression analyses including both log frequency and log^2^ frequency. For many languages, the frequency effect levels off at high frequencies, resulting in a deviation from the linear regression. To capture this deviation, Balota et al. [28] proposed to add a quadratic frequency component to the equation (parabolic functions can be described by polynomials of degree 2). Table 3 lists the percentages of variance accounted for when both log freq and log^2^ freq are entered into the regression. This table shows (1) that the SUBTL_logCHR index is slightly better than the other measures, and (2) that character frequencies outperform word frequencies. A stepwise multiple regression analysis indicated that no other frequency measure reached significance once SUBTL_logCHR was entered.

**Table 3:** The percentages of variance in RTs accounted for by each of eight available frequency measures (four word frequency measures and four character frequency measures), for 2289 single character words in a word naming task.

N = 2289	Word frequency				Character frequency			
	SUBTL_logW	SUBTL_ logW-CD	LCSMCS_logW	LCMC_logW	SUBTL_logCHR	SUBTL_ logCHR-CD	LCSMCS_logCHR	CCL_ logCHR
Log	28.3	28.4	22.9	30.1	32.0	32.6	31.2	29.9
log+log^2^	29.7	28.7	23.7	32.0	33.0	32.6	33.9	33.0

Because we also wanted to have information about two-character words, we additionally looked for such a data set. This was found in a series of lexical decision experiments published by Myers et al. [29] or presented at the International Conference on the Mental Lexicon in Canada in 2004 and 2006. In these experiments participants had to decide between real words and made-up combinations of first and second characters matched on frequency and complexity. The various experiments provided information about 206 words, all of which were compounds (162 nouns and 44 verbs). With this data set, we compared how much of the variance was explained by our word frequencies and how much was explained by the other word frequency measures. First, we excluded 5 words which were region-specific (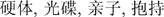) or related to a special culture (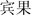 = Bingo, name of a gambling game). The data from one participant were excluded as well, because the reaction times were too long (beyond 3 standard deviations from the mean reaction time of the other participants). For the other participants, reaction times beyond 3 standard deviations from the mean were excluded (overall loss of observations <2%). RTs were calculated on correct trials only.

SUBTLEX-CH covered 200 of the 201 words, while LCSMCS covered 189 words, and LCMC covered 199 words. We ran the correlation and regression analyses on the 187 words that were covered by all frequency measures. Correlations between the RTs and the frequencies were -.654 for SUBTL_logW, -.654 for SUBTL_logW-CD, -.370 for LCSMCS_logW, -.522 for LCMC_logW. Intriguingly, when we added the character frequencies, we also found a correlation of -.325 for SUBTL_logCHR of the first character in the word but not of the second character (all ps<0.01).

We used a stepwise multiple regression analysis to investigate whether a combination of frequency measures explained extra variance in the RT data. The results showed that SUBTL_logW-CD was the most significant predictor (p<0.001), explaining 42.8% of the variance. LCMC word frequency explained 2.8% in addition (p<0.001). Once the effects of these two frequencies were taken into account, the frequency of the first character no longer reached significance. Table 4 lists the percentages of variance of RT explained by each frequency measures (log), when we also included the variance explained by log^2^. It shows that SUBTLEX-CH clearly outperforms LCMC and even more LCSMCS.

**Table 4:** The percentages of variance in RT accounted for by each of the different frequency measures, for two-character words in the visual lexical decision task.

N = 187	Word frequency			
	SUBTL_ logW	SUBTL_ logW-CD	LCSMCS_logW	LCMC_ logW
Log	42.7	42.8	13.7	27.3
log+log^2^	44.8	43.9	13.7	27.9

**Figure 4:**
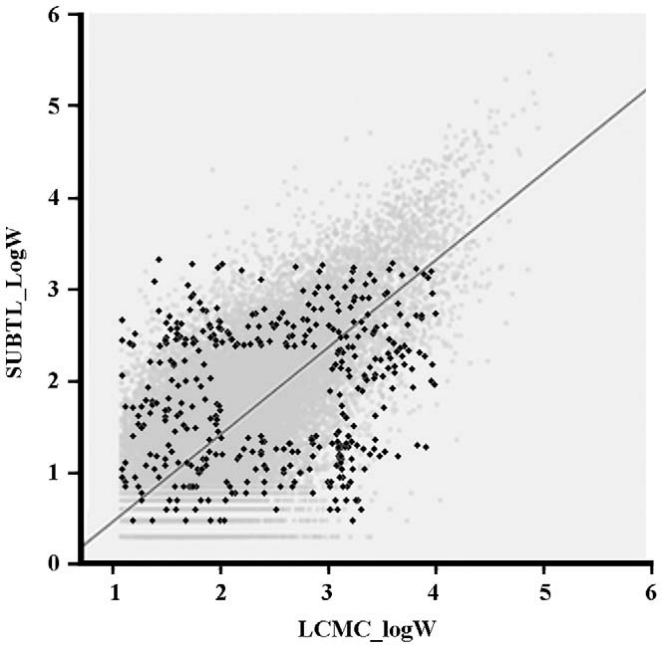
The light gray points on the background represent the 28,336 two-character words included in both SUBLTEX-CH and LCMC, together with their log10 frequencies; the black diamonds represent the 400 words selected for the lexical decision validation study.

Given that we could only find a limited data set with two-character words in the literature, we decided to run an extra small-scale lexical decision validation experiment with 400 words and 400 non-words. The stimulus words were selected in such a way that they pitted SUBTL_logW against LCMC (the two best measures in the previous analysis). To give each frequency measure the best possible chance, we selected words that were high/low on them and that did not correlate much with the other frequency measure (Figure 4).

A convenience sample of 12 Chinese-speaking participants living in Belgium and France took part in the lexical decision task (mean age 28.8 years; range 25–38 years, 7 males and 5 females). All participants were native Chinese speakers and had at least 16 years of education (all finished university). A trial started with a central fixation stimulus for 500 ms, followed by the word or non-word presented at the center of a computer screen until the participant responded or for a maximum of 2000 ms. Participants were asked to press as quickly and accurately as possible with the left index finger on the c-key of the keyboard or with right index finger on the m-key, to decide whether the stimulus corresponded to an existing Chinese word or was a made-up combination of two characters (left-right hand response was counterbalanced between participants). The non-words were created from the characters used in the set of words stimuli, by recombining the first and second characters in non-word character pairs. A blank screen of 200 ms was presented between the response and the start of the next trial. Optional breaks were possible after every 80 trials. The task took about half an hour.

To analyze the RT data, we started with some basic cleaning procedures. First, we excluded the words that were not correctly recognized by at least half of the participants. This was the case for 6 of the 400 words (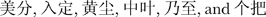). We also excluded one non-word because it was seen by more than half of the participants as a word. For each participant, trials with a wrong response and/or with reaction times beyond 2.5 standard deviations from the mean were marked as missing (8.4% of the observations). Because of the small number of participants, the missing data were replaced with estimates based on the mean RT to the word plus the mean RT of the participant minus the overall mean RT of the experiment. In this way, the word processing times were not distorted by missing values from slow or fast participants. Despite the small participant sample, the split-half correlation for the thus obtained data set was .7 (attenuated for length).

The correlations between the RTs and the frequencies for the remaining 394 words were: −.496 for SUBTL_logW, −.502 for SUBTL_logW-CD, and −.427 for LCMC_logW. When we added the character frequencies of the first and the second characters, we also obtained a significant correlation for the frequency of the first character (r =  −.133, p<0.01), but not of the second character. Because LCSMCS covered 322 of the 394 words, we further calculated the correlation for this measure (LCSMCS_logW), which was −.305. Further comparisons using William's method (for dependent correlations) showed that the SUBTLEX frequency measures perform significantly better than the LCSMCS measures in explaining the variance in the RTs (r_SUBTL_logW: RT_ = −.497, r_LCSMCS_logW: RT_ = −.305, r_ SUBTL_logW: LCSMCS_logW_ = .167; t(319) = − 3.07, p<0.005; r_SUBTL_logW-CD: RT_ = −.495, r_LCSMCS_logW: RT_ = −.305, r_ SUBTL_logW-CD: LCSMCS_logW_ = .192; t(319) = −3.07, p<0.005) and tend to be better than LCMC (r_SUBTL_logW: RT_ = −.496, r_LCMC_logW: RT_ = −.427, r_ SUBTL_logW: LCMC_logW_ = .191; t(391) = −1.28, p<.05, one-tailed; r_SUBTL_logW-CD: RT_ = −.502, r_LCMC_logW: RT_ = −.427, r_ SUBTL_logW-CD: LCMC_logW_ = .225, t(391) = −1.43, p<.04, one-tailed).

A stepwise regression showed, consistent with the results obtained from the previous data set, that SUBTL_logW-CD was the most significant frequency predictor (p<0.001), explaining 25.2% of the variance. LCMC_logW explained 10.5% in addition (p<0.001). The frequency of the first character no longer was significant once the effects of these two measures were taken into account. Table 5 lists the percentages of variance of RT explained by each frequency measures.

**Table 5:** The percentages of variance in RT accounted for by each of the different frequency measures, for two-character words in our lexical decision task.

N = 394	Word frequency			
	SUBTL_ logW	SUBTL_ logW-CD	LCSMCS_logW*	LCMC_ logW
Log	24.6	25.2	9.3	18.3
log+log^2^	24.6	25.3	10.2	19.1

*N = 322.

Interestingly, when we looked at the individual data, we saw that four participants had a higher correlation with the LCMC frequencies than with the SUBTLEX frequencies. These tended to be the older participants.

Given that the non-words were based on the set of characters used in the word stimuli, we also ran a regression analysis on the 399 non-words, to investigate the potential roles of character frequency in Chinese non-word rejection performance. The results showed that neither the frequency of the first character nor that of the second character explained any variance in the RTs to the non-words.

## Discussion

We presented and tested new frequency measures for Mandarin Chinese based on subtitles. Our results confirm that these word frequencies are a good estimate of daily language exposure and capture much of the variance in word processing efficiency. The subtitle measures are of the same quality as the existing ones for single-character words (at least in the naming task tested) and outperform the existing frequency indices for two-character words. The finding that character frequencies predict RTs for single-character words better than the frequencies of these characters as independent words is in line with the proposal that in Chinese characters play a key role in the lexical structure of words and the access to them [22]. However, for two-character words, although there is a negative correlation between RTs and the frequencies of the first characters, we saw that character frequencies no longer contributed to the variance in RTs once the word frequencies were taken into account in the multiple regression analyses. In all likelihood, the observed correlation between the RTs and the first character frequencies was an artifact of the positive correlation between charword frequencies and character frequencies.

For two-character words, our results show that the LCSMCS word frequencies are of limited value, probably because they are based on a small part of the corpus (2 million words, mainly from the *People's Daily*; see [8]). In contrast, the more recently collected LCMC measures may provide interesting additional information to SUBTLEX-CH. In English and French, it has also been found that word frequencies based on written texts explain a few percentages of extra variance in visual lexical decision data to those based on film subtitles, even though the written frequencies themselves are inferior to the subtitle frequencies. Further research will have to indicate whether word frequencies based on written texts also explain additional variance in the performance on auditory word recognition tasks, given that subtitles arguably provide a better estimate of spoken word use than written text materials.

As in other languages, the contextual diversity measure does slightly better than the frequency counts, urging researchers to make more use of this measure. On the other hand, the difference seems to be rather small in the various analyses we ran, suggesting that not much information will be lost if researchers in Chinese continue to use the familiar frequency counts rather than the CD-measure. Finally, to our knowledge, the present database is the first to include information about the different syntactic roles of the words. Although we did not make use of this information in the analyses reported here, it is our conviction that this will be of great interest for future researchers.

Because our research was covered by a non-commercial grant (see the acknowledgments), we can give free access to the outcome for research purposes (see Files S1, or alternatively go to http://expsy.ugent.be/subtlex-ch). As indicated above, there are three different frequency files (SUBTLEX-CH-CHR, SUBTLEX-CH-WF, and SUBTLEX-CH-WF_PoS) containing the character frequencies (both frequencies based on counts and frequencies based on CD), the word frequencies, and information about the frequencies of the different syntactic roles words play.

## Supporting Information

Table S1 Labels used in the PKU PoS system.
(0.03 MB DOC)

Files S1 SUBTLEX is a zipped file including three files (SUBTLEX-CH-WF, SUBTLEX-CH-CHR, SUBTLEX-CH-WF_PoS) providing word and character frequency measures based on a corpus of film subtitles (33.5 million words or 46.8 million characters).
(1.76 MB ZIP)
