# Supplementary material for: SUBTLEX-CH: Chinese Word and Character Frequencies Based on Film Subtitles
Source: PLoS One. 2010 Jun 2;5(6):e10729. doi: 10.1371/journal.pone.0010729 (PMC2880003; doi:10.1371/journal.pone.0010729)
Supplement: Table S1 — Labels used in the PKU PoS system. (0.03 MB DOC) [file pone.0010729.s001.doc]

**Supporting Table 1. Labels used in the PKU PoS system.**

| a adjective  ad adjective as adverbial  ag adjective morpheme  an adjective with nominal function  b non-predicate adjective  c conjunction  d adverb  dg adverb morpheme  e interjection  f directional locality  g morpheme  h prefix  i idiom  j abbreviation  k suffix  l fixed expressions  m numeral  mg numeric morpheme  n common noun  ng noun morpheme  nr personal name | ns place name  nt organization name  nx nominal character string  nz other proper noun  o onomatopoeia  p preposition  q classifier  r pronoun  rg pronoun morpheme  s space word  t time word  tg time word morpheme  u auxiliary  v verb  vd verb as adverbial  vg verb morpheme  vn verb with nominal function  w symbol and non-sentential punctuation  x unclassified items  y modal particle  z descriptive |
| --- | --- |
